# Beta-Thalassemia and Male Infertility: Unraveling the Oxidative Stress Connection—An Up-to-Date Review

**DOI:** 10.3390/diagnostics14242789

**Published:** 2024-12-12

**Authors:** Christos Roidos, Christos-Alexandros Batakoias, Evangelos N. Symeonidis, Aris Kaltsas, Vasileios Tzikoulis, Georgios Tsampoukas, Chara Tsiampali, Natalia Palapela, Athanasios Zachariou, Nikolaos Sofikitis, Fotios Dimitriadis

**Affiliations:** 1 First Department of Urology, Faculty of Medicine, School of Health Sciences, Aristotle University of Thessaloniki, 54124 Thessaloniki, Greece; drchriroid22@gmail.com (C.R.); bill1996tziko@gmail.com (V.T.); 2 Medical Faculty, Aristotle University, 54124 Thessaloniki, Greece; chrisbatakoias07@gmail.com; 3 Department of Urology II, European Interbalkan Medical Center, 55535 Thessaloniki, Greece; evansimeonidis@gmail.com; 4 Third Department of Urology, Attikon University Hospital, School of Medicine, National and Kapodistrian University of Athens, 12462 Athens, Greece; ares-kaltsas@hotmail.com; 5 Department of Urology, Homerton University Hospital, London E9 6SR, UK; tsampoukasg@gmail.com; 6 Independent Researcher, 55131 Thessaloniki, Greece; x.tsiampali@gmail.com; 7 Medical Faculty, Medical University of Sofia, 1431 Sofia, Bulgaria; nataliapalapela21@gmail.com; 8 Department of Urology, Faculty of Medicine, School of Health Sciences, University of Ioannina, 45110 Ioannina, Greece; zahariou@otenet.gr (A.Z.); nsofikit@uoi.gr (N.S.)

**Keywords:** beta-thalassemia, male infertility, oxidative stress, reactive oxygen species, antioxidants

## Abstract

Background/Objectives: Beta-thalassemia (BTH), a genetic disorder resulting from beta-globin gene mutations, affects over 1.5 million people globally. The disorder’s multifactorial impact on male fertility, particularly through oxidative stress (OS), warrants focused study. This review examines the mechanisms of OS in TM, its implications for male infertility, and the potential of antioxidant therapies to mitigate fertility challenges. Methods: A non-systematic review was conducted using the PubMed, Cochrane, and Medscape databases, focusing on studies on beta-thalassemia (BTH), erectile dysfunction (ED), hormonal alterations, and OS. Studies were screened based on relevance, language, and topic, with 71 articles meeting the inclusion criteria after removing duplicates. Results: The findings reveal that OS, exacerbated by iron overload from regular blood transfusions, is significantly associated with impaired sperm quality and fertility in patients with TM. Iron toxicity affects gonadotropin levels, reduces sperm quality, and contributes to hypogonadism. Additionally, antioxidant therapies show promise in reducing OS-induced sperm damage, though efficacy is limited by a lack of robust clinical trials. Conclusions: OS plays a considerable role in male infertility among patients with TM, primarily through iron-induced sperm damage and hormonal disruptions. While antioxidant therapies may offer a partial remedy, further research is necessary to understand OS’s mechanisms in TM and develop effective fertility treatments. This review highlights the need for personalized antioxidant approaches to improve reproductive outcomes in this population.

## 1. Introduction

Thalassemia major (TM) affects more than 1,500,000 people worldwide [1]. According to the Thalassemia International Federation, only about 200,000 patients with TM are alive and registered as receiving regular treatment around the world [2]. The term thalassemia is derived from the Greek thalassa (sea) and haima (blood). Beta-thalassemia (BTH) includes three main forms: (i) TM (homozygous), variably referred to as “Cooley’s Anemia” and “Mediterranean Anemia”; (ii) Thalassemia Intermedia (mosaicism); and (iii) Thalassemia Minor (heterozygous), also called “beta-thalassemia carrier”, “beta-thalassemia trait”, or “heterozygous beta-thalassemia”.

The cause of this disease is a mutation in the beta-globin gene, which leads to reduced amounts of beta-globin. TM is usually suspected in infants younger than two years of age with severe microcytic anemia, mild jaundice, and hepatosplenomegaly. Thalassemia intermedia presents at a later age with similar but milder clinical findings. Carriers are usually asymptomatic but sometimes may have mild anemia [3].

The therapeutic options for BTH consist of blood transfusions, along with iron chelating agents, deferoxamine (DFX), and deferiprone (DFP), to treat the subsequent iron overload, and bone marrow transplantation (BMT), which is the only definite treatment currently available. Following the latter therapeutic option, the iron burden slowly decreases, but the rate of decline is accelerated by regular phlebotomy or iron chelation therapy after BMT [4,5]. Gene therapy is a breakthrough treatment option, having earned a Nobel prize in 2020, but it is still used on a small scale [6].

Erectile dysfunction (ED) and infertility are significant complications in male patients with TM. Recent studies indicate that ED affects approximately 50–70% of patients with TM, primarily due to hypogonadism, iron-induced oxidative stress (OS), and vascular damage. Infertility rates are also notably high, with up to 60% of male patients with TM experiencing impaired reproductive capacity. These conditions are exacerbated by iron overload from chronic transfusions and inadequate chelation therapy, which disrupts gonadotropin secretion and damages sperm quality [7,8].

OS is defined as a condition that reflects an imbalance between the systemic manifestation of reactive oxygen species (ROS) and the ability of the biological system to readily detoxify (antioxidant defenses) reactive intermediates or to repair the resulting damage [9,10]. According to the latest version of the EAU Guidelines on Male Infertility, OS plays a crucial role in male infertility by compromising sperm quality, function, and integrity. It can cause sperm DNA damage and reduce DNA integrity, leading to poor embryo development, miscarriage, and infertility [11]. Spermatozoa are especially susceptible to OS and have limited DNA repair abilities. This stress is often linked to poor lifestyle choices (e.g., smoking) and environmental factors, suggesting that antioxidants and lifestyle changes could reduce DNA fragmentation and improve sperm quality [12,13]. However, these findings lack support from randomized controlled trials (RCTs). While ROS can be measured through various assays like chemiluminescence, standardized testing methods for ROS are unavailable [9]. Thus, routine ROS measurement should remain experimental until validated by RCTs.

The WHO defines infertility as the inability of a sexually active, non-contracepting couple to achieve pregnancy in one year [14]. It affects 15% of couples globally. The male factor participates in 50% of infertile couples, whereas it is exclusively responsible for 20–30% of the cases [15,16]. Male infertility, rather than a personal medical condition, is a social problem that represents a growing threat in the already increased number of unwillingly childless couples in Western societies.

This review explores the association between b-thalassemia and male infertility, focusing on the role of OS.

## 2. Materials and Methods

PubMed, Cochrane, and Medscape were used as search engines. The following keywords—alone and in combinations—were used: “beta-thalassemia”, “erectile dysfunction”, “hormonal alterations”, “male infertility”, “beta-thalassemia treatment”, “oxidative stress”, and “semen parameters”. The titles and abstracts extracted from the electronic searches were scrutinized, duplicates were removed, and the full manuscripts of relevant citations that met the search-predefined selection criteria were obtained. We considered English studies examining the association of erectile dysfunction and male infertility with BTH to be eligible for inclusion. Furthermore, studies evaluating the role of OS in the pathogenesis of erectile dysfunction and male infertility associated with beta-thalassemia were deemed qualified for inclusion in this review. Non-English-language articles and studies on topics unrelated to BTH and male infertility were excluded. After examining the complete manuscripts, the final studies were incorporated into our final analysis based on co-author consensus.

Following deduplication, two reviewers screened the abstracts and titles of identified records for eligibility. The full texts were retrieved and screened, and the study characteristics were extracted in a standardized form. Study characteristics, participant features, type of biopsy specimen, reported primary and secondary outcomes of each study, and study inclusion and exclusion criteria were extracted from each study.

### Data Collection and Risk of Bias Assessment

Data from the studies included in this review were extracted using a pre-defined data extraction form. Subsequently, an additional member of the review team verified the extracted data to ensure reliability and completeness. In cases where multiple articles were derived from the same study population, only the most recent report was included.

Evidence synthesis was performed using a narrative format. Due to the quality and heterogeneity of the included studies, a quantitative synthesis of the evidence was not performed. Owing to variations in targets and methodological approaches among the included studies, statistical pooling and a meta-analysis of the results were considered inappropriate.

**Figure 1:**
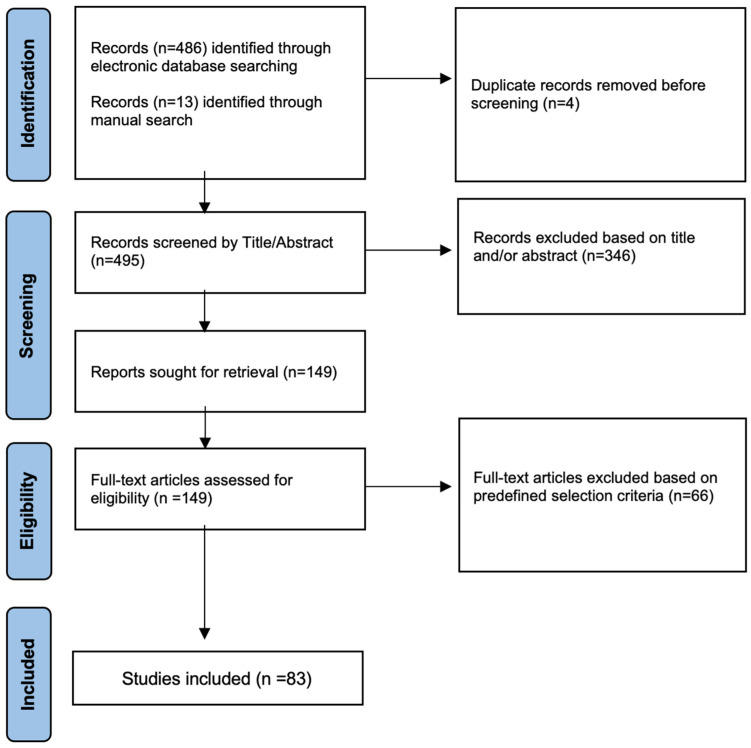
A flow diagram of the studies included in the narrative review.

Of the 499 studies retrieved from the electronic search, 83 were included in the final narrative synthesis. Figure 1 demonstrates a flow diagram of the studies included in this up-to-date review.

## 3. Deciphering the Interplay Between Oxidative Stress, Beta-Thalassemia, and Male Infertility

### 3.1. Semen Parameters

Iron’s role in mediating ROS formation in biological systems is well known. The harmful effects of ROS on the sperm membrane, its structural components, and its nucleus are also well documented [17]. Studies have reported that 34% to 87% of patients had abnormal semen profiles [18,19,20]. Studies of sperm concentrations in eight patients with BTH reported severe oligoasthenospermia [18]. In another study, 50% of the patients with TM submitted to long-lasting treatment with DFX had normal sperm counts, motility, and morphology [21]. At the same time, the remaining patients had oligospermia and/or asthenospermia. Seminal parameters may change over time, and DFX chelation therapy may harm spermatogenesis and/or sperm function [21].

A small study of men with transfusion-dependent BTH reported elevated seminal plasma iron concentrations in five of six men, with concentrations approximately 5–10 times higher than the reference range [22]. Of the five men, semen parameters were abnormal in four [22]. Males with BTH are at risk of infertility due to germ cell loss [23]. Iron overload also induces excessive free radicals, leading to sperm quality impairment, while iron is a local catalyzer of ROS [24,25]. The first MRI study on testes (2017) showed that in patients with transfusion-dependent BTH, MRI T2 values are lower versus those in healthy controls and correlate with serum ferritin; iron overload-related testicular damage explains infertility in males without hypogonadotropic hypogonadism [26]. In a small study of 16 fully grown patients with thalassemia intermedia, a significant correlation between total sperm count, motility, ferritin, and folate levels was observed. However, the possible clinical utility of these findings is hindered by some limitations, referring to sample size and heterogeneity [27].

Perera et al. reported that DNA damage is also higher in men with BTH than in normozoospermic controls [28]. However, as far as DNA sperm damage is concerned, a study including 20 male patients with fully pubertal BTH, 10 normozoospermic and 10 azoospermic, failed to confirm the findings of Perera et al. [28]. DFI starting from a baseline of <30%, which is considered a critical value, above which pregnancy rates decrease, did not differ significantly after antioxidant treatment. However, other parameters of sperm damage, such as the number of middle pieces with defects, the sperm deformity index (SDI), and the teratozoospermic index increased significantly. This was considered a potential side effect of treatment with L-carnitine and N-acetyl cysteine in the studied patients with BTM. Another finding of this study was the correlation of anemia severity with azoospermia, pointing out a possible role of genetic modifiers, which control the rate of hemolysis and the phenotypic severity of BTH, to the development of late-onset male hypogonadism [27].

A study carried out by Soliman et al. found a statistically significant improvement in semen parameters, along with hormonal improvements, acutely following blood transfusion in males aged 17–32 with fully pubertal BTM [29]. Regarding the effects of chelation therapy on semen parameters, abnormal sperm count and motility were associated with lower ferritin levels in patients with BTH compared to those with normozoospermia, indicating a possible association of semen parameter impairment with intensive chelation therapy [30].

### 3.2. Hormonal Alterations

In transfusion-dependent patients with BTH, iron toxicity begins when the stores of proteins that bind iron in tissue and blood are depleted. This leads to transfer in the reticuloendothelial system, and then enters the parenchyma, causing substantial oxidative damage to various organs [31,32]. Studies have shown that endocrine glands are susceptible to this toxicity, and one in four patients under the age of 12 presents with problems in one or more glands [33]. These problems are associated with hypogonadism, hypothyroidism, adrenal insufficiency, hypoparathyroidism, developmental disorders, disturbances in the glycemic profile, and bone disease, with the participation rate of each gland varying between studies [33].

In many studies of transfusion-dependent BTH, the highest percentage of endocrinopathies involved disorders of the hypothalamus–pituitary–gonadal (HPG) axis [23,34,35,36,37,38,39,40,41,42,43]. Disorders during adolescence, such as ED with or without infertility, were present in a percentage ranging from 51% to 80% of patients [44].

It has been reported that the mean level of ferritin in patients with HPG dysfunction was significantly higher in patients with defective function than in patients with normal function [45]. In another study, it was found that patients with ferritin levels greater than 2500 ng/mL are 2.75 times more likely (95% CI 1.38–5.48) to have hypogonadism than patients with levels less than 1000 ng/mL [46]. Investigators demonstrated this through magnetic resonance imaging of pituitary gland atrophy in transfusion-dependent patients with BTH and hemochromatosis [36] and showed that signal intensity reduction in the anterior lobe of the pituitary gland correlated with the serum ferritin level and the severity of pituitary dysfunction [47]. Serum testosterone levels were inversely proportional to the pituitary gland’s iron levels [48]. Additionally, in a small study of seven patients with transfusion-dependent BTM and two control individuals, the pituitary size and levels of gonadotropic hormones were reduced in the individuals in the first group compared to those in the control group [22]. Also, it was found that the pituitary size and fertility of patients undergoing persistent iron chelation therapy were maintained at more satisfactory levels [22].

Furthermore, studies report that in cases of iron overload in the body, its deposition occurs in the gonadotropic cells of the pituitary gland [49,50]. It is mentioned that levels of FSH and LH are affected in patients with thalassemia; however, GnRH stimulation tests demonstrate that LH is more sensitive to iron and shows a greater reduction in states of iron overload than FSH [19]. In a study of 95 patients with thalassemia and a control group of 50 individuals, statistically significant reductions in leptin, ghrelin, and sexual hormones were observed [23].

Sporadically, the testicular iron overload takes over the pituitary involvement, leading to hypergonadotropic hypogonadism [51,52,53,54,55,56]. In a study involving 95 patients, of whom 52 were transfusion-dependent and 43 had undergone allogeneic stem cell transplantation, the percentage of hypogonadism was 32.6% (31 patients) in the first group and 30.8% (16 patients) in the second, with no statistically significant difference between them [51]. However, while in the first group, all 31 patients exhibited hypogonadotropic hypogonadism, in the second group, 4 out of the 16 patients (25%) presented with hypergonadotropic hypogonadism [51].

In another study, the gonadotropin levels in the patients with BTH-delayed puberty were compared with the constitutional delayed puberty group. There were no significant differences in the basal hormonal levels. Still, the response to GnRH administration was meager in the β-thalassemia-delayed puberty group compared to the response of the constitutional delayed puberty group. On the other hand, no significant difference was noted between the basal and the peak levels of LH and FSH between the β-thalassemia normal puberty group and the control group [1,45]. A similar study showed that human chorionic gonadotropin (hCG)-stimulated testosterone production was normal in all individuals with constitutional delayed puberty. In contrast, it was reduced in 42% of patients with delayed puberty due to BTH [26,55].

Data from a recent study by Soliman et al. showed that anemia plays a crucial role in the pathogenesis of hypogonadism in transfusion-dependent patients with BTH. Significant improvements in sperm parameters and sexual hormones were observed after packed red blood cell (PRBC) transfusion [29].

### 3.3. Erectile Dysfunction

Sexual dysfunction may occur in most cases subsequently to hypogonadism, but sometimes it is primary with detrimental repercussions for erection [57]. For a patient with thalassemia already undergoing the stressful demands of therapy, the problem of impotence can provoke a state of pervasive depression that tends to cloud every aspect of the patient’s life. The self-confidence of these patients is severely affected.

Iron deposits are associated with interstitial siderosis of the interlunar trabeculae of the corpora cavernosa and subsequently with fibrotic replacement. This consequent reduction and/or loss of ejaculation hinders erection regardless of the patient’s hormonal status, implying a possible non-hormonal mechanism [58]. A retrospective cohort found that even b-thalassemia heterozygotes had a 4.6-fold risk for developing erectile dysfunction when compared to non-thalassemic controls [59].

### 3.4. Fertility Potential

Paternity in men with thalassemia, even for those with normal or near-normal spermatogenesis, is less common than pregnancy in women with thalassemia [60]. Some patients with hypogonadism and damage to the hypothalamic–pituitary axis have spontaneous sexual maturation and intact fertility potential [18].

In males suffering from azoospermia or asthenospermia and asking for fertility treatment, spermatogenesis may be induced by combination therapy with hCG and human menopausal gonadotrophin (hMG) intramuscularly or subcutaneously [61]. A study of the outcomes of assisted reproductive technologies (ARTs) in men with BTH found that therapy with gonadotropin +/− GnRH could only induce spermatogenesis in six of nineteen men, with no spontaneous and only two successful IVF-ICSI pregnancies attained [62].

Males with BTH are generally at a higher risk for infertility due to progressive germ cell loss [23]. A comparative study pointed out that fertility is reduced in males—between 16 and 41 years old—who had undergone hematopoietic stem cell transplant (*N* = 43) compared to those treated with transfusion and chelation (*N* = 52). The prevalence of hypogonadism (32%) did not differ among the groups above, indicating that male fertility is a result of non-hormonal causes [51].

Moreover, the advent of micromanipulation techniques such as intracytoplasmic sperm injection (ICSI) has improved conception rates [63]. The selection of sperm for this procedure should be undertaken with much care to prevent predisposing these embryos to risks of further genetic disease. Therefore, appropriate genetic counseling is of paramount importance [64].

### 3.5. Interconnection of Hypogonadism, Erectile Dysfunction, and Infertility

The triad of hypogonadism, ED, and infertility in males with BTM is indeed a complex clinical challenge. The damage to the HPG axis caused by iron overload, a hallmark of BTM, leads to low testosterone (T), which significantly affects sexual function and spermatogenesis. Low T levels are often associated with ED, a common issue in patients with BTM, due to the compromised NO synthesis required for proper vascular function [51,58].

Additionally, OS plays a significant role in exacerbating these conditions. Chronic transfusion therapy, a cornerstone of BTM management, contributes to endothelial damage and worsens vascular dysfunction, further complicating ED [58]. Psychological distress arising from sexual dysfunction often worsens both ED and infertility [51]. From a therapeutic perspective, testosterone replacement therapy (TRT) is the first line of treatment for hypogonadism, aiming to restore sexual function, spermatogenesis, and overall hormonal balance. However, this treatment must be carefully monitored, particularly for patients with cardiovascular concerns [58].

As far as infertility is concerned, gonadotropins, such as hCG and FSH, may be used to stimulate spermatogenesis in men with residual testicular function [51]. Phosphodiesterase type 5 inhibitors (PDE5is) like sildenafil can also effectively manage ED by improving penile blood flow [58]. Moreover, by reducing OS, antioxidants are emerging as an adjunctive treatment to support both hormone- and ED-specific therapies [51,58].

This integrated therapeutic approach aims to address the multifaceted challenges faced by patients with BTM, optimizing both reproductive and sexual health.

## 4. Beta-Thalassemia and Oxidative Stress: Current Knowledge and Recent Advances

Patients with BTH have been shown to have higher levels of OS compared to healthy controls. Molecular and cellular damage is more severe in these patients, which can be attributed both to the insufficiency of their antioxidant defenses—despite the increased OS—the degradation of the unstable hemoglobin, and the accumulation of ROS due to iron overload caused by repetitive blood transfusion [65,66].

Because of the prolonged life expectancy and improvement in the quality of life, the desire of patients with thalassemia to attain reproductive capacity and have biological children becomes a significant task. The studies mentioned above highlight the hormonal alterations occurring in patients with BTM as well as the semen parameter anomalies and their effect on fertility. The observed subfertility in this subgroup of patients is multifactorial and could not be attributed solely to hormonal causes [51]. OS—regardless of its cause—has previously been associated with male infertility and semen parameter anomalies [67].

ROS are unstable oxygen molecules that are derived from an incomplete reduction in molecular oxygen at the end stage of oxidative phosphorylation [68]. Under normal circumstances, ROS play a key role in sperm’s chromatin condensation, spermatozoa activation, and spermatozoa number regulation via apoptosis [69]. Extra modifications occur during spermatozoa transit through the epididymis, leading to their maturation [70]. When the ROS concentration exceeds the spermatozoa’s total antioxidant capacity (TAC), OS occurs. Spermatozoa are vulnerable to OS [71]. Genetic causes, varicoceles, genitourinary infections and trauma, cancer and treatments against it, obesity, nutrient deficiency, smoking, drugs, pollution, high temperature, and radiation lead to OS, which causes protein damage, lipid peroxidation, DNA damage, and apoptosis, resulting in sperm damage and infertility [15,16]. A lower TAC has been associated with impaired sperm motility and infertility [70]. OS has been further associated with the impairment of all semen parameters and microscopic defects, and it is mostly associated with impaired sperm motility [70]. Higher ROS concentrations have been associated with semen parameter disorders, such as oligoasthenospermia (OAT). A cut-off concentration value of 91.95 Relative Light Units (RLUs) has been proposed as a diagnostic tool for OS and as a prognosis index in assisted reproductive technology (ART) procedures [72].

Various mechanisms, such as OS, a lack of antioxidant defenses, and the sole impact of chelating agents on DNA, have been proposed to explain the DNA damage observed in patients with BTH [28,73]. In another study, the seminal antioxidant pattern of iron-overloaded patients with BTM indicated the hyperactivation of the enzymatic free radical scavengers, which could be explained as a compensatory mechanism for possible high levels of reactive oxygen species. Furthermore, the increase in seminal lipoperoxidation suggested OS in the semen of these patients, and it could have contributed to the impairment of sperm motility [74].

The lack of standardized protocols and the complex interactions between antioxidants often lead to inconsistent results across studies. Even though combining different antioxidants may theoretically provide a broader benefit, the individual effects of each antioxidant remain challenging to isolate. These issues continue to complicate the clinical application of antioxidant therapy in male infertility. In the study by Li et al. (2022), a network meta-analysis highlighted the variable effectiveness of antioxidants in improving sperm quality and pregnancy rates for men with idiopathic infertility, underscoring the challenges of assessing the individual contributions of different antioxidant components [75]. Similarly, Barbonetti et al. (2024) conducted a network meta-analysis that focused on the effect of antioxidants on semen parameters in men with OAT, finding significant variability in the outcomes based on the specific antioxidants used. While antioxidant therapy holds potential, these studies emphasize that the results are often inconsistent and may depend on patient-specific factors and the antioxidant regimen employed [76]. These findings highlight the need for further research to address the complexities surrounding antioxidant therapy in male infertility with an emphasis on creating standardized treatment protocols to improve clinical outcomes.

Iron overload-induced OS is a key mechanism for male infertility in BTH; however, ferroptosis—a regulated form of cell death driven by iron accumulation and lipid peroxidation—may represent an additional pathway. Ferroptosis is characterized by glutathione depletion, lipid peroxidation, and cellular dysfunction, processes that could exacerbate testicular damage and impair spermatogenesis [77]. Emerging evidence suggests that ferroptosis markers, such as reduced glutathione peroxidase 4 (GPX4) activity, are associated with reproductive injury under iron-overloaded conditions. Addressing ferroptosis through targeted antioxidants or inhibitors could complement existing oxidative stress therapies, offering novel therapeutic avenues for mitigating male infertility in this population [78].

**Figure 2:**
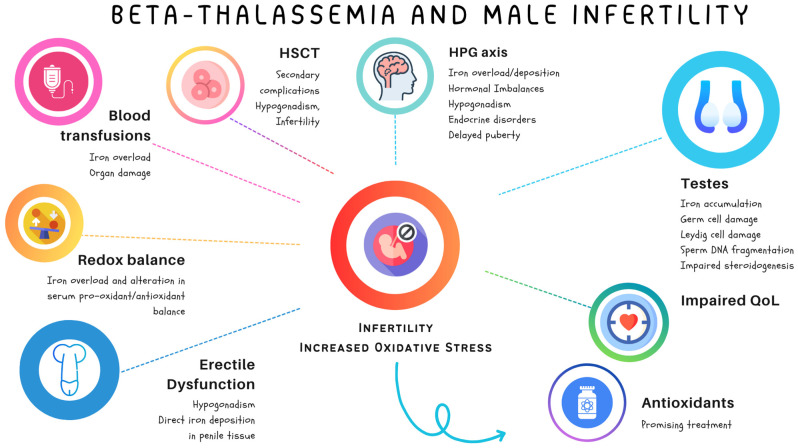
Oxidative stress and antioxidant therapy in beta-thalassemia-related infertility.

In the context of BTH and male infertility, antioxidants may alter the oxidative state and restore OS-induced sperm damage. In recent years, several authors have shed light on their potential therapeutic benefits and risks and highlighted the nuanced effects of antioxidant administration on male infertility. While antioxidants like vitamins C and E, coenzyme Q10, and selenium can mitigate oxidative stress and improve sperm parameters, a critical balance must be maintained [79]. Over-supplementation or inappropriate use may disrupt redox homeostasis, leading to detrimental effects on sperm quality and function [80]. Hence, physicians dealing with patients with BTH-related infertility should be aware of the importance of administering these compounds while maintaining a redox equilibrium. The complex interplay of BTH, male infertility, and OS is schematically illustrated in Figure 2.

## 5. Future Perspectives and Promising Areas of Research

First, future research should focus on personalized antioxidant therapy, considering individual oxidative stress levels and genetic predispositions, particularly in patients with BTH who are prone to elevated oxidative stress. Second, although antioxidants constitute a promising approach, an accurate and standardized approach in therapy has yet to be seen. To date, several questions remain unanswered about the exact dosage and specific type of nutrient combinations to be administered. Third, longitudinal studies examining the long-term effects of antioxidant supplementation on fertility outcomes are essential. As previously suggested, the implications of antioxidant therapy in the context of emerging health challenges, such as COVID-19, also warrant further exploration to understand their potential role in mitigating virus-induced oxidative damage and subsequent male infertility. By tailoring antioxidant treatment to the specific needs of patients, we can enhance therapeutic efficacy while minimizing adverse effects, paving the way for the improved management of male infertility in BTH. More robust research in the field will also clarify the optimal diagnostic and therapeutic pathway of OS-induced infertility in this group of patients [81].

An emerging 21st-century threat must be highlighted, considering the risk of germ cell loss and the deleterious effects of OS on the gonads of patients with BTH. An increased paternal age has dramatically changed the andrological landscape during the past few years. Informing patients with BTH about the harmful effects of an advanced father’s age on reproductive outcomes remains challenging, albeit necessary. Physicians should be aware of this relationship and guide them effectively [82,83].

Focusing only on a cure for BTH can come at a future cost. Noteworthily, patients with BTH may face a progressive loss of spermatogonia before or during the disease course.

Aside from malignancies, testicular tissue (TT) cryopreservation has gained ground even for non-malignant diseases like BTH. Those at risk of becoming infertile should be offered TT banking. This is of great importance for patients with BTH who will eventually end up receiving myeloablative conditioning therapy and hematopoietic stem cell transplantation (HSCT). The latter represents the only viable curative option for BTH, albeit with well-known side effects and a risk for stem cell pool loss. Also, pre-pubertal patients unable to cryopreserve their semen samples should discuss testicular tissue cryostorage if they desire to produce offspring in the future. In addition, adult males need to be advised to proceed with sperm cryopreservation, thus increasing the chances of having biological children in the future. Lastly, future studies should elaborately examine fertility preservation techniques in patients with BTH.

## 6. Limitations

Our narrative review is subject to several limitations. Although it thoroughly explores up-to-date knowledge, our study is prone to publication bias inherent to its non-systematic nature. Another limitation arises from the scarcity of data reporting on male infertility and its association with OS in patients with BTH. The literature on the interplay mentioned above is rather heterogeneous and outdated, consisting of reviews, retrospective studies with short follow-ups, small samples, and a lack of extensive data.

Nonetheless, despite being one of the few studies that evaluated the association of factors influencing BTH, subfertility, and OS, the present review raises many questions about using antioxidants in treating OS-induced infertility in patients with BTH. Although their role is well established, it needs to be more generalizable to a broader BTH population, and randomized controlled trials can pave the way for more definite conclusions. Likewise, we also acknowledge the need for further impactful research in the field, which will address identified gaps more systematically.

## 7. Conclusions

Beta-thalassemia has been associated with impaired male fertility, and the role of OS is, to some extent, evident. It would be logical to assume an association between BTH and infertility, with oxidative stress being the mediating mechanism. However, these data remain controversial, and currently available studies do not focus on achieving child-bearing. There is an evident lack of high-quality studies that have fertility as their primary outcome while examining the role of OS in achieving the outcome and the effects of an antioxidant regimen. Moreover, further research should be conducted with the aim of clarifying mechanisms and leading to potential new therapeutic strategies as far as the fertility of these patients is concerned.

## References

[1] Chatterjee R., Katz M. (2001). Evaluation of gonadotrophin insufficiency in thalassemic boys with pubertal failure: Spontaneous versus provocative test. J. Pediatr. Endocrinol. Metab..

[2] Cappellini M.D., Cohen A., Eleftheriou A., Piga A., Porter J., Taher A. (2008). Guidelines for the Clinical Management of Thalassaemia.

[3] Galanello R., Origa R. (2010). Beta-thalassemia. Orphanet J. Rare Dis..

[4] Angelucci E., Muretto P., Lucarelli G., Ripalti M., Baronciani D., Erer B., Galimberti M., Annibali M., Giardini C., Gaziev D. (1998). Treatment of iron overload in the “ex-thalassemic”. Report from the phlebotomy program. Ann. N. Y. Acad. Sci..

[5] Giardini C., Galimberti M., Lucarelli G., Polchi P., Angelucci E., Baronciani D., Gaziev D., Erer B., La Nasa G., Barbanti I. (1995). Desferrioxamine therapy accelerates clearance of iron deposits after bone marrow transplantation for thalassaemia. Br. J. Haematol..

[6] Huang J., Zhang Y., Liang L., Wu J., Li X., Ye Z., Lin B., Ma J., Su L., Ye Q. (2023). Gene Therapy of Transfusion-Dependent β-Thalassemia Patients with Quick Engraftment of Reinfused Hematopoietic Stem Cells: An Investigator-Initiated Trial of KL003. Blood.

[7] Kaltsas A., Zikopoulos A., Dimitriadis F., Sheshi D., Politis M., Moustakli E., Symeonidis E.N., Chrisofos M., Sofikitis N., Zachariou A. (2024). Oxidative Stress and Erectile Dysfunction: Pathophysiology, Impacts, and Potential Treatments. Curr. Issues Mol. Biol..

[8] McCabe M.P., Sharlip I.D., Lewis R., Atalla E., Balon R., Fisher A.D., Laumann E., Lee S.W., Segraves R.T. (2016). Incidence and Prevalence of Sexual Dysfunction in Women and Men: A Consensus Statement from the Fourth International Consultation on Sexual Medicine 2015. J. Sex. Med..

[9] Symeonidis E.N., Evgeni E., Palapelas V., Koumasi D., Pyrgidis N., Sokolakis I., Hatzichristodoulou G., Tsiampali C., Mykoniatis I., Zachariou A. (2021). Redox Balance in Male Infertility: Excellence through Moderation-”Muepsilontaurhoomicronnu ἄrhoiotasigmatauomicronnu”. Antioxidants.

[10] Saalu L.C. (2010). The incriminating role of reactive oxygen species in idiopathic male infertility: An evidence based evaluation. Pak. J. Biol. Sci..

[11] Gkeka K., Symeonidis E.N., Tsampoukas G., Moussa M., Issa H., Kontogianni E., Almusafer M., Katsouri A., Mykoniatis I., Dimitriadis F. (2023). Recurrent miscarriage and male factor infertility: Diagnostic and therapeutic implications. A narrative review. Cent. Eur. J. Urol..

[12] Kaltsas A. (2023). Oxidative Stress and Male Infertility: The Protective Role of Antioxidants. Medicina.

[13] Kaltsas A., Zachariou A., Dimitriadis F., Chrisofos M., Sofikitis N. (2024). Empirical Treatments for Male Infertility: A Focus on Lifestyle Modifications and Medicines. Diseases.

[14] (2010). WHO Laboratory Manual for the Examination and Processing of Human Semen.

[15] Hirsh A. (2003). Male subfertility. BMJ.

[16] Hirsh A. (2003). ABC of subfertility: Male subfertility. BMJ.

[17] Aitken R.J., Gordon E., Harkiss D., Twigg J.P., Milne P., Jennings Z., Irvine D.S. (1998). Relative impact of oxidative stress on the functional competence and genomic integrity of human spermatozoa. Biol. Reprod..

[18] Jensen C.E., Abdel-Gadir A., Cox C., Tuck S.M., Wonke B. (1996). Sperm concentrations and quality in beta-thalassaemia major. Int. J. Androl..

[19] Papadimas J., Mandala E., Pados G., Kokkas B., Georgiadis G., Tarlatzis B., Bontis J., Sinakos Z., Mantalenakis S. (1996). Pituitary-testicular axis in men with beta-thalassaemia major. Hum. Reprod..

[20] Katz M., De Sanctis V., Vullo C., Wonke B., Ughi M., Pinamonti A., Sprocati M., Gamberini M.R., Bagni B., Andò S., Brancati C. (1995). Spermatogenesis in Patients with β-Thalassaemia major and intermedia. Endocrine Disorders in Thalassemia: Physiopathological and Therapeutical Aspects.

[21] De Sanctis V., Perera D., Katz M., Fortini M., Gamberini M.R. (2008). Spermatozoal DNA damage in patients with B thalassaemia syndromes. Pediatr. Endocrinol. Rev..

[22] Singer S.T., Killilea D., Suh J.H., Wang Z.J., Yuan Q., Ivani K., Evans P., Vichinsky E., Fischer R., Smith J.F. (2015). Fertility in transfusion-dependent thalassemia men: Effects of iron burden on the reproductive axis. Am. J. Hematol..

[23] De Sanctis V., Soliman A.T., Elsedfy H., Di Maio S., Canatan D., Soliman N., Karimi M., Kattamis C. (2017). Gonadal dysfunction in adult male patients with thalassemia major: An update for clinicians caring for thalassemia. Expert Rev. Hematol..

[24] Elsedfy H., De Sanctis V., Ahmed A.Y., Mohamed N.R., Arafa M., Elalfy M.S. (2018). A pilot study on sperm DNA damage in beta-thalassemia major: Is there a role for antioxidants?. Acta Biomed..

[25] Gabrielsen J.S., Lamb D.J., Lipshultz L.I. (2018). Iron and a Man’s Reproductive Health: The Good, the Bad, and the Ugly. Curr. Urol. Rep..

[26] Chen M.J., Peng S.S., Lu M.Y., Yang Y.L., Jou S.T., Chang H.H., Chen S.U., Lin D.T., Lin K.H. (2018). Effect of iron overload on impaired fertility in male patients with transfusion-dependent beta-thalassemia. Pediatr. Res..

[27] De Sanctis V., Candini G., Giovannini M., Raiola G., Katz M. (2011). Abnormal seminal parameters in patients with thalassemia intermedia and low serum folate levels. Pediatr. Endocrinol. Rev..

[28] Perera D., Pizzey A., Campbell A., Katz M., Porter J., Petrou M., Irvine D.S., Chatterjee R. (2002). Sperm DNA damage in potentially fertile homozygous beta-thalassaemia patients with iron overload. Hum. Reprod..

[29] Soliman A., Yasin M., El-Awwa A., Osman M., de Sanctis V. (2012). Acute effects of blood transfusion on pituitary gonadal axis and sperm parameters in adolescents and young men with thalassemia major: A pilot study. Fertil. Steril..

[30] De Sanctis V., Borsari G., Brachi S., Govoni M., Carandina G. (2008). Spermatogenesis in young adult patients with beta-thalassaemia major long-term treated with desferrioxamine. Georgian Med. News.

[31] Hoffbrand A.V., Wonke B. (1997). Iron chelation therapy. J. Intern. Med. Suppl..

[32] Britton R.S., Ramm G.A., Olynyk J., Singh R., O‘Neill R., Bacon B.R. (1994). Pathophysiology of iron toxicity. Adv. Exp. Med. Biol..

[33] Carsote M., Vasiliu C., Trandafir A.I., Albu S.E., Dumitrascu M.C., Popa A., Mehedintu C., Petca R.C., Petca A., Sandru F. (2022). New Entity-Thalassemic Endocrine Disease: Major Beta-Thalassemia and Endocrine Involvement. Diagnostics.

[34] De Sanctis V., Soliman A., Mohamed Y. (2013). Reproductive health in young male adults with chronic diseases in childhood. Pediatr. Endocrinol. Rev..

[35] Srisukh S., Ongphiphadhanakul B., Bunnag P. (2016). Hypogonadism in thalassemia major patients. J. Clin. Transl. Endocrinol..

[36] Soliman A.T., elZalabany M.M., Ragab M., Abdel Fattah M., Hassab H., Rogol A.D., Ansari B.M. (2000). Spontaneous and GnRH-provoked gonadotropin secretion and testosterone response to human chorionic gonadotropin in adolescent boys with thalassaemia major and delayed puberty. J. Trop. Pediatr..

[37] Hekmatnia A., Radmard A.R., Rahmani A.A., Adibi A., Khademi H. (2010). Magnetic resonance imaging signal reduction may precede volume loss in the pituitary gland of transfusion-dependent beta-thalassemic patients. Acta Radiol..

[38] Cianciulli P., Del Poeta G., De Sanctis V. (1986). Reperti istopatologici gonadici ed ipofisari nell’anemia di Cooley. La Maturazione Sessualenella β-Talassemia Major.

[39] Vullo C., De Sanctis V., Katz M., Wonke B., Hoffbrand A.V., Bagni B., Torresani T., Tolis G., Masiero M., Di Palma A. (1990). Endocrine abnormalities in thalassemia. Ann. N. Y. Acad. Sci..

[40] Lee K.T., Lim S.L., Goh A.S. (2020). Prevalence of endocrine complications in transfusion dependent thalassemia in Hospital Pulau Pinang: A pilot study. Med. J. Malaysia.

[41] Bordbar M., Bozorgi H., Saki F., Haghpanah S., Karimi M., Bazrafshan A., Zekavat O.R. (2019). Prevalence of endocrine disorders and their associated factors in transfusion-dependent thalassemia patients: A historical cohort study in Southern Iran. J. Endocrinol. Investig..

[42] Ehsan L., Rashid M., Alvi N., Awais K., Nadeem O., Asghar A., Sajjad F., Fatima M., Qidwai A., Hussain S. (2018). Clinical utility of endocrine markers predicting myocardial siderosis in transfusion dependent thalassemia major. Pediatr. Blood Cancer.

[43] Lambrou G.I., Samartzi A., Vlachou E., Papassotiriou I., Geronikolou S.A., Kanaka-Gantenbein C., Chrousos G.P., Kattamis A. (2021). Genotypic and Clinical Analysis of a Thalassemia Major Cohort: An Observational Study. Adv. Exp. Med. Biol..

[44] De Sanctis V., Elsedfy H., Soliman A.T., Elhakim I.Z., Pepe A., Kattamis C., Soliman N.A., Elalaily R., El Kholy M., Yassin M. (2016). Acquired Hypogonadotropic Hypogonadism (AHH) in Thalassaemia Major Patients: An Underdiagnosed Condition?. Mediterr. J. Hematol. Infect. Dis..

[45] Al-Rimawi H.S., Jallad M.F., Amarin Z.O., Al Sakaan R. (2006). Pubertal evaluation of adolescent boys with beta-thalassemia major and delayed puberty. Fertil. Steril..

[46] Belhoul K.M., Bakir M.L., Saned M.S., Kadhim A.M., Musallam K.M., Taher A.T. (2012). Serum ferritin levels and endocrinopathy in medically treated patients with beta thalassemia major. Ann. Hematol..

[47] Sparacia G., Iaia A., Banco A., D‘Angelo P., Lagalla R. (2000). Transfusional hemochromatosis: Quantitative relation of MR imaging pituitary signal intensity reduction to hypogonadotropic hypogonadism. Radiology.

[48] Noetzli L.J., Panigrahy A., Mittelman S.D., Hyderi A., Dongelyan A., Coates T.D., Wood J.C. (2012). Pituitary iron and volume predict hypogonadism in transfusional iron overload. Am. J. Hematol..

[49] Bergeron C., Kovacs K. (1978). Pituitary siderosis. A histologic, immunocytologic, and ultrastructural study. Am. J. Pathol..

[50] Kontogeorgos G., Handy S., Kovacs K., Horvath E., Scheithauer B.W. (1996). The Anterior Pituitary in Hemochromatosis. Endocr. Pathol..

[51] Rostami T., Mohammadifard M.A., Ansari S., Kiumarsi A., Maleki N., Kasaeian A., Aghamahdi F., Rad S., Ghavamzadeh A. (2020). Indicators of male fertility potential in adult patients with beta-thalassemia major: A comparative study between patients undergone allogeneic stem cell transplantation and transfusion-dependent patients. Fertil. Res. Pract..

[52] Raiola G., Galati M.C., De Sanctis V., Caruso Nicoletti M., Pintor C., De Simone M., Arcuri V.M., Anastasi S. (2003). Growth and puberty in thalassemia major. J. Pediatr. Endocrinol. Metab..

[53] Low L.C. (2005). Growth of children with beta-thalassemia major. Indian J. Pediatr..

[54] Al-Rimawi H.S., Jallad M.F., Amarin Z.O., Obeidat B.R. (2005). Hypothalamic-pituitary-gonadal function in adolescent females with beta-thalassemia major. Int. J. Gynaecol. Obstet..

[55] Soliman A.T., Nasr I., Thabet A., Rizk M.M., El Matary W. (2005). Human chorionic gonadotropin therapy in adolescent boys with constitutional delayed puberty vs those with beta-thalassemia major. Metabolism.

[56] Oerter K.E., Kamp G.A., Munson P.J., Nienhuis A.W., Cassorla F.G., Manasco P.K. (1993). Multiple hormone deficiencies in children with hemochromatosis. J. Clin. Endocrinol. Metab..

[57] Rund D., Rachmilewitz E. (1995). Thalassemia major 1995: Older patients, new therapies. Blood Rev..

[58] Lombardo T., Giammusso B., Frontini V., D‘Arpa S., Pafumi C., Caruso S. (2000). Thalassaemic men affected by erectile dysfunction treated with transurethral alprostadil: Case report. Hum. Reprod..

[59] Chen Y.G., Lin T.Y., Lin C.L., Dai M.S., Ho C.L., Kao C.H. (2015). Risk of erectile dysfunction in transfusion-naive thalassemia men: A nationwide population-based retrospective cohort study. Medicine.

[60] Aessopos A., Karabatsos F., Farmakis D., Katsantoni A., Hatziliami A., Youssef J., Karagiorga M. (1999). Pregnancy in patients with well-treated beta-thalassemia: Outcome for mothers and newborn infants. Am. J. Obstet. Gynecol..

[61] Skordis N., Petrikkos L., Toumba M., Hadjigavriel M., Sitarou M., Kolnakou A., Skordos G., Pangalou E., Christou S. (2004). Update on fertility in thalassaemia major. Pediatr. Endocrinol. Rev..

[62] Bajoria R., Chatterjee R. (2011). Hypogonadotrophic hypogonadism and diminished gonadal reserve accounts for dysfunctional gametogenesis in thalassaemia patients with iron overload presenting with infertility. Hemoglobin.

[63] Origa R., Piga A., Quarta G., Forni G.L., Longo F., Melpignano A., Galanello R. (2010). Pregnancy and beta-thalassemia: An Italian multicenter experience. Haematologica.

[64] Sakkas D., Manicardi G., Bizzaro D., Bianchi P.G. (2000). Possible consequences of performing intracytoplasmic sperm injection (ICSI) with sperm possessing nuclear DNA damage. Hum. Fertil..

[65] Fatima T., Khan S., Khan M.M., Kamran R., Uddin M.W., Sohrab S. (2023). Oxidative Stress in Beta-thalassemia Patients: Role of Enzymatic and Non-enzymatic Modulators. Protein Pept. Lett..

[66] Fibach E., Dana M. (2019). Oxidative Stress in beta-Thalassemia. Mol. Diagn. Ther..

[67] Agarwal A., Virk G., Ong C., du Plessis S.S. (2014). Effect of oxidative stress on male reproduction. World J. Men’s Health.

[68] Berg J., Tymoczko J., Stryer L. (2002). Biochemistry.

[69] Alahmar A.T., Sengupta P. (2021). Impact of Coenzyme Q10 and Selenium on Seminal Fluid Parameters and Antioxidant Status in Men with Idiopathic Infertility. Biol. Trace Elem. Res..

[70] El-Taieb M.A., Herwig R., Nada E.A., Greilberger J., Marberger M. (2009). Oxidative stress and epididymal sperm transport, motility and morphological defects. Eur. J. Obstet. Gynecol. Reprod. Biol..

[71] Ahmadi S., Bashiri R., Ghadiri-Anari A., Nadjarzadeh A. (2016). Antioxidant supplements and semen parameters: An evidence based review. Int. J. Reprod. Biomed..

[72] Agarwal A., Sharma R.K., Sharma R., Assidi M., Abuzenadah A.M., Alshahrani S., Durairajanayagam D., Sabanegh E. (2014). Characterizing semen parameters and their association with reactive oxygen species in infertile men. Reprod. Biol. Endocrinol..

[73] De Sanctis V., Vullo C., Katz M., Wonke B., Nannetti C., Bagni B. (1988). Induction of spermatogenesis in thalassaemia. Fertil. Steril..

[74] Carpino A., Tarantino P., Rago V., De Sanctis V., Siciliano L. (2004). Antioxidant capacity in seminal plasma of transfusion-dependent beta-thalassemic patients. Exp. Clin. Endocrinol. Diabetes.

[75] Li K.P., Yang X.S., Wu T. (2022). The Effect of Antioxidants on Sperm Quality Parameters and Pregnancy Rates for Idiopathic Male Infertility: A Network Meta-Analysis of Randomized Controlled Trials. Front. Endocrinol.

[76] Barbonetti A., Tienforti D., Castellini C., Giulio F.D., Muselli M., Pizzocaro A., Vena W., Baroni M.G., Pivonello R., Isidori A.M. (2024). Effect of antioxidants on semen parameters in men with oligo-astheno-teratozoospermia: A network meta-analysis. Andrology.

[77] Prabhune N.M., Ameen B., Prabhu S. (2024). Therapeutic potential of synthetic and natural iron chelators against ferroptosis. Naunyn Schmiedebergs Arch. Pharmacol..

[78] Liu Y., Cao X., He C., Guo X., Cai H., Aierken A., Hua J., Peng S. (2022). Effects of Ferroptosis on Male Reproduction. Int. J. Mol. Sci..

[79] Dimitriadis F., Tsounapi P., Zachariou A., Kaltsas A., Sokolakis I., Hatzichristodoulou G., Symeonidis E.N., Kotsiris D., Gabales M.R., Vlachopoulou E. (2021). Therapeutic Effects of Micronutrient Supplements on Sperm Parameters: Fact or Fiction?. Curr. Pharm. Des..

[80] Dimitriadis F., Symeonidis E.N., Tsounapi P., Kaltsas A., Hatzichristodoulou G., Sokolakis I., Zachariou A., Takenaka A., Sofikitis N. (2021). Administration of Antioxidants in Infertile Male: When it may have a Detrimental Effect?. Curr. Pharm. Des..

[81] Kalfas T., Kaltsas A., Symeonidis E.N., Symeonidis A., Zikopoulos A., Moustakli E., Tsiampali C., Tsampoukas G., Palapela N., Zachariou A. (2023). COVID-19 and Male Infertility: Is There a Role for Antioxidants?. Antioxidants.

[82] Kaltsas A., Moustakli E., Zikopoulos A., Georgiou I., Dimitriadis F., Symeonidis E.N., Markou E., Michaelidis T.M., Tien D.M.B., Giannakis I. (2023). Impact of Advanced Paternal Age on Fertility and Risks of Genetic Disorders in Offspring. Genes.

[83] Kaltsas A., Zikopoulos A., Vrachnis D., Skentou C., Symeonidis E.N., Dimitriadis F., Stavros S., Chrisofos M., Sofikitis N., Vrachnis N. (2024). Advanced Paternal Age in Focus: Unraveling Its Influence on Assisted Reproductive Technology Outcomes. J. Clin. Med..

